# Supplementary material for: Heated Tobacco Products and Nicotine Pouches: A Survey of People with Experience of Smoking and/or Vaping in the UK
Source: Int J Environ Res Public Health. 2021 Aug 22;18(16):8852. doi: 10.3390/ijerph18168852 (PMC8391640; doi:10.3390/ijerph18168852)
Supplement: Supplementary file 1 [file ijerph-18-08852-s001.zip › ijerph-1295968-supplementary.pdf]

Heated tobacco products and nicotine pouches: A survey of people with experience of smoking and/or vaping in the UK.

Leonie S. Brose, Máirtín McDermott, Ann McNeill

Supplemental material

Table S1. Bivariate associations between ever use of, and interest in HTPs, ever use of nicotine pouches and socio-demographics and smoking & vaping status, n=3883 for ever use, n=1199 for interest

	HTP ever use[1]		HTP interest		Nicotine Pouches ever use	
	OR (95 CI)	p	OR (95 CI)	p	OR (95 CI)	p
**Gender**						
Male	1.00 (0.77 - 1.30)	0.98	0.84 (0.66-1.05)	0.128	**1.41 (1.03 - 1.92)**	**0.030**
Female	Ref		Ref		Ref	
**Age**						
18-24	**8.40 (3.70 - 19.09)**	**<0.001**	**3.62 (2.05-6.39)**	**<0.001**	**9.61 (3.31 - 27.89)**	**<0.001**
25-34	**8.68 (3.97 - 18.98)**	**<0.001**	**4.51 (2.81-7.26)**	**<0.001**	**11.89 (4.3 - 32.88)**	**<0.001**
35-44	**6.28 (2.86 - 13.77)**	**<0.001**	**3.67 (2.32-5.80)**	**<0.001**	**7.98 (2.87 - 22.21)**	**<0.001**
45-54	2.28 (0.99 - 5.25)	0.054	**1.95 (1.26-3.01)**	**0.003**	2.55 (0.86 - 7.56)	0.093
55-65	**2.37 (1.00 - 5.59)**	**0.049**	**1.98 (1.27-3.08)**	**0.003**	1.86 (0.58 - 5.97)	0.296
Over 65	Ref		Ref		Ref	
**Ethnicity**						
Not white	**1.87 (1.29 - 2.70)**	**0.001**	1.21 (0.81-1.79)	0.352	**2.1 (1.39 - 3.18)**	**<0.001**
White	Ref		Ref		Ref	
**Education**						
Some university	**3.04 (2.28 - 4.03)**	**<0.001**	**1.34 (1.07-1.68)**	**0.011**	**3.37 (2.39 - 4.75)**	**<0.001**
No university	Ref		Ref		Ref	
**Region**						
Greater London	**2.27 (1.69 - 3.05)**	**<0.001**	**1.38 (1.02-1.87)**	**0.038**	**2.80 (1.98 - 3.96)**	**<0.001**
Wales	0.39 (0.14 - 1.07)	0.066	0.86 (0.49-1.49)	0.586	0.32 (0.08 - 1.30)	0.110
Scotland	0.66 (0.37 - 1.17)	0.157	0.74 (0.48-1.15)	0.176	1.43 (0.84 - 2.43)	0.184
Northern Ireland	0.62 (0.19 - 1.99)	0.422	1.85 (0.85-4.00)	0.119	1.02 (0.32 - 3.29)	0.973

	HTP ever use[1]		HTP interest		Nicotine Pouches ever use	
	**OR (95 CI)**	**p**	**OR (95 CI)**	**p**	**OR (95 CI)**	**p**
England excl London	Ref		Ref		Ref	
**Smoking /Vaping**						
Not smoking or vaping	0.71 (0.44-1.16)	0.176	**0.31 (0.21-0.46)**	**<0.001**	1.14 (0.68-1.90)	0.621
Smoking and vaping	**3.01 (2.29-4.11)**	**<0.001**	**1.60 (1.19-2.15)**	**0.002**	**3.38 (2.38-4.81)**	**<0.001**
Vaping only	0.73 (0.44-1.19)	0.206	0.76 (0.55-1.04)	0.090	0.68 (0.36-1.29)	0.238
Smoking only	Ref		Ref		Ref	
